# The pivotal role of inflammatory factors in glaucoma: a systematic review

**DOI:** 10.3389/fimmu.2025.1577200

**Published:** 2025-05-23

**Authors:** Bin Lin, Dongkan Li

**Affiliations:** ^1^ Xiamen Eye Center and Eye Institute of Xiamen University, School of Medicine, Xiamen, China; ^2^ Clinical Research Center for Eye Diseases, Xiamen, Fujian, China; ^3^ Xiamen Key Laboratory of Ophthalmology, Xiamen, Fujian, China; ^4^ Fujian Key Laboratory of Corneal and Ocular Surface Diseases, Xiamen, Fujian, China; ^5^ Xiamen Key Laboratory of Corneal and Ocular Surface Diseases, Xiamen, Fujian, China; ^6^ Translational Medicine Institute of Xiamen Eye Center of Xiamen University, Xiamen, Fujian, China

**Keywords:** glaucoma, inflammatory factors, pathogenesis, neuroinflammation, therapeutic targets

## Abstract

Glaucoma, a leading cause of irreversible vision loss, is becoming more prevalent with the aging population, burdening patients, families, and society. In the past, the role of inflammatory factors in its pathogenesis was overlooked. This systematic review, based on a PubMed search and strict screening of 61 articles, selected 19 for in-depth analysis. It was found that multiple inflammatory factors like Tumor Necrosis factor alpha (TNF – α), Interleukin-6 (IL-6), and Interleukin-1 (IL–1) are abnormal in glaucoma patients’ intraocular fluid. They impact trabecular meshwork function, damage retinal ganglion cells, and activate the complement system. Other factors such as Vascular Endothelial Growth Factor (VEGF) and Monocyte Chemoattractant Protein-1 (MCP-1) also contribute to the disease process. Based on these findings, emerging therapeutic strategies for glaucoma may include biological agents targeting specific inflammatory mediators, multitarget anti-inflammatory approaches, and personalized interventions guided by inflammatory biomarker profiling. However, critical challenges such as blood-retinal barrier penetration limitations, systemic immunosuppression risks, and technical hurdles in gene therapy delivery require further investigation. This systematic review synthesizes current evidence to inform clinical decision-making regarding inflammatory biomarker monitoring while identifying key knowledge gaps in ocular immunomodulation. The findings underscore the necessity for translational studies bridging preclinical models with clinical applications, ultimately aiming to optimize therapeutic outcomes for glaucoma patients worldwide.

## 1 Introduction

Glaucoma is a major eye disease that causes irreversible vision impairment worldwide (1). It is estimated that the global prevalence of glaucoma in the population aged 40 to 80 is 3.5%. With the increase in the number and proportion of the elderly in the population, it is projected that by 2040, 111.8 million people will suffer from glaucoma (2). Glaucoma not only severely impairs patients’ vision and reduces their quality of life but also increases the medical burden on families and society (3), posing a significant challenge to global public health.

In recent years, scholars have achieved substantial results in the research on inflammatory factors in the intraocular fluid of glaucoma patients. Studies have found that the levels of multiple inflammatory factors in the intraocular fluid of glaucoma patients are abnormal (4–7). For example, the increase in pro-inflammatory factors such as vascular endothelial growth factor (VEGF), interleukin-6 (IL-6), and interleukin-8 (IL-8) accelerates the occurrence and development of glaucoma. These changes indicate that the inflammatory response is crucial in the pathogenesis of glaucoma, providing new insights into exploring the disease process of glaucoma.

Given this research progress, this clinical research review is of great significance. It can help clinicians quickly master the cutting-edge knowledge in this field, provide theoretical support for the diagnosis and treatment of glaucoma, and achieve early diagnosis and treatment by monitoring inflammatory factors. In addition, it can also clarify the deficiencies of existing research, guide future scientific research, promote innovation in glaucoma treatment, and contribute to improving the prognosis of glaucoma patients worldwide.

## 2 Methods

We employed a Boolean logic search strategy with a timeframe extending from the database’s inception to January 2025. The search strategy was as follows: ((“Glaucoma”[Mesh] OR glaucoma[Title/Abstract] OR glaucomatous[Title/Abstract]) AND ((“Staging”[Mesh] OR staging[Title/Abstract] OR stage[Title/Abstract] OR “Classification”[Mesh] OR classification[Title/Abstract] OR classify[Title/Abstract]) AND ((“Intraocular”[Mesh] OR intraocular[Title/Abstract] OR ocular[Title/Abstract]) AND (“Inflammatory factors”[Mesh] OR “Inflammatory cytokines”[Mesh] OR “Inflammatory mediators”[Mesh] OR “Inflammatory markers”[Mesh] OR “Inflammatory proteins”[Mesh] OR inflammatory factor*[Title/Abstract] OR inflammatory cytokine*[Title/Abstract] OR inflammatory mediator*[Title/Abstract] OR inflammatory marker*[Title/Abstract] OR inflammatory protein*[Title/Abstract] OR cytokine*[Title/Abstract] OR mediator*[Title/Abstract] OR marker*[Title/Abstract] OR protein*[Title/Abstract])). The search engine used was PubMed, which yielded 61 relevant articles.

The exclusion criteria for the literature are as follows:

**Objective:** To screen studies that experimentally validate the molecular mechanisms of inflammatory mediators in the pathogenesis of glaucoma.
**Exclusion Criteria:**
**Irrelevant Research Content**
Studies involving other organ systems or non-glaucomatous ocular diseases.
Studies focus on non-inflammatory mechanisms.
Studies involving other organ or ophthalmic diseases, with no more than three mentions of the term “glaucoma”.
**Lack of Mechanistic Research**
Studies that only describe the changes in the levels of inflammatory mediators without exploring the molecular mechanisms.
Studies with fewer than three substantive references to inflammatory mediators or without experimental verification.
**Methodological Limitations**
Technical reports or device-development-oriented studies without exploring biological mechanisms.
Non-empirical literature, such as commentaries, letters, and isolated case reports.
**Insufficient Causal Verification**
Correlational analysis without combined functional experiments.
Studies that do not use causal verification methods.

**Figure 1:**
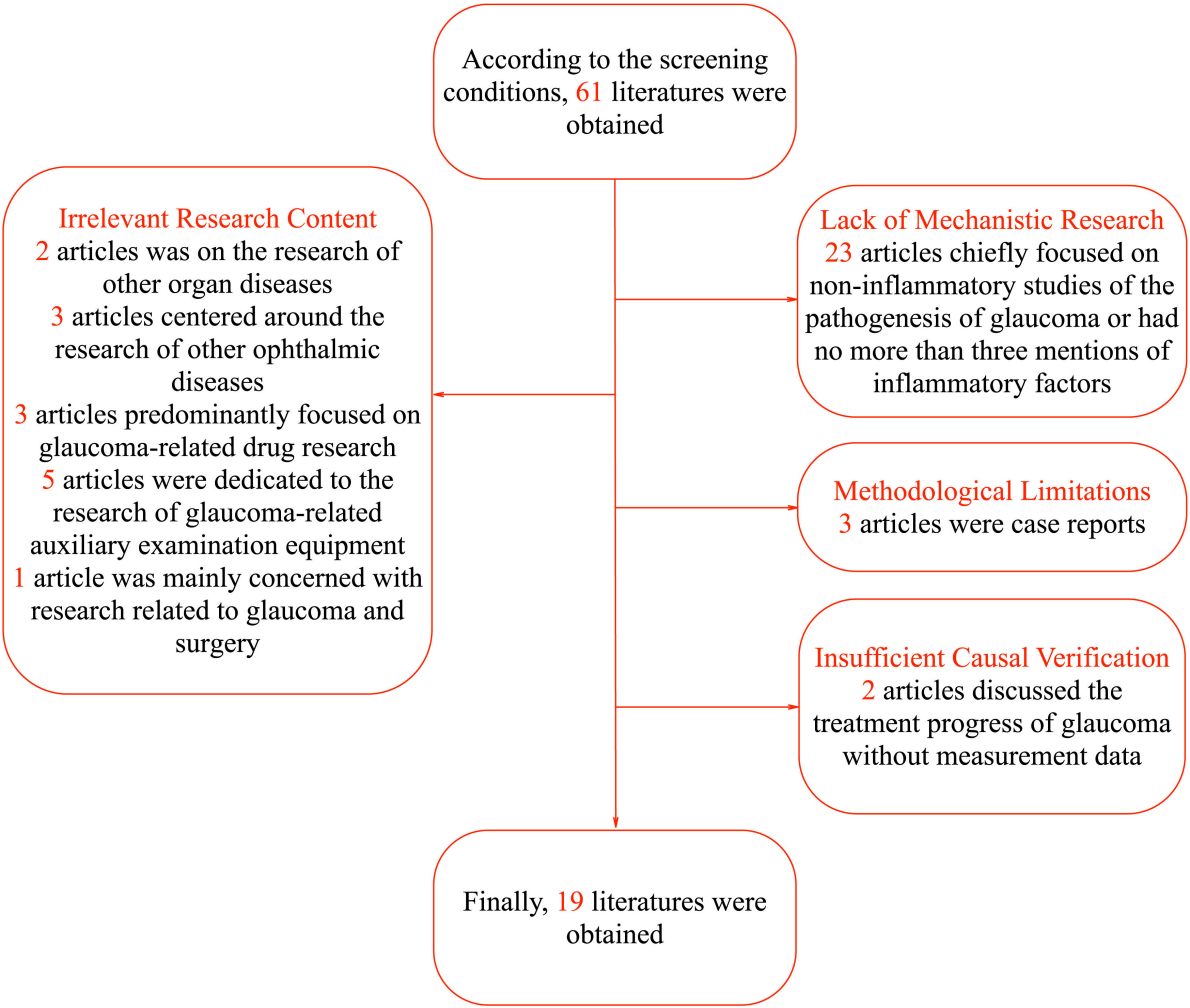
Literature screening process diagram.

Regarding the literature screening process, 42 articles were excluded for not meeting the research criteria. Specifically, the focus of 2 articles was on the research of other organ diseases with no more than three mentions of the term “glaucoma”, and 3 articles centered around the research of other ophthalmic diseases with no more than three mentions of the term “glaucoma”. Three articles predominantly focused on glaucoma-related drug research, 5 articles were dedicated to the research of glaucoma-related auxiliary examination equipment, and 1 article was mainly concerned with research related to glaucoma and surgery. In terms of lack of mechanistic research, 23 articles chiefly focused on non-inflammatory studies of the pathogenesis of glaucoma or had no more than three mentions of inflammatory factors. Considering methodological limitations, 3 articles were case reports. Regarding insufficient causal verification, 2 articles discussed the treatment progress of glaucoma without measurement data. In terms of lack of mechanistic research, 23 articles were excluded. Some of these articles focused on non-inflammatory aspects of glaucoma pathogenesis, which deviated from our focus on inflammatory mediators. Others had no more than three mentions of inflammatory factors, indicating a lack of in-depth exploration of their role. Thus, they did not meet the requirements for this review. Finally, a total of 19 articles were included, namely Havens S.J. et al. (8), Zhang L et al. (9), Musciano A.R. et al. (10), Mrugacz M et al. (5), Kapuganti R.S. et al. (11), Chen J et al. (12), Simcoe M.J. et al. (13), Chou T.H. et al. (14), Jonas J.B. et al. (4), Donkor N et al. (15), Nezu N et al. (6), Wang H.N. et al. (16), Azbukina N.V. et al. (17), Yun S et al. (18), Frolov M.A. et al. (19), Cao L et al. (20), Elshahaly M et al. (21), Wang CY et al. (22), Reinehr S et al. (7). The specific process is shown in **Figure 1**. The particular content of the finally selected articles is shown in **Table 1**.

**Table 1:** Summary of key findings on inflammatory mechanisms and biomarkers in glaucoma pathogenesis.

Author(s)	Journal	Key Findings
Havens S.J. et al.	Dev Ophthalmol	Retinal ischemia triggers inflammation, and inflammatory cells secrete pro-angiogenic factors such as VEGF, promoting neovascularization, blocking aqueous outflow, and leading to increased intraocular pressure (IOP). Inflammation also affects myofibroblast function and extracellular matrix synthesis, accelerating angle closure and IOP elevation.
Zhang L et al.	FEBS J	lncRNA can regulate the expression of inflammatory factors such as IL and TNF, for example, by acting as ceRNA to influence the mRNA expression of inflammatory factors, exacerbating inflammatory damage in glaucoma. Targeting relevant lncRNAs may offer therapeutic potential for glaucoma.
Musciano A.R. et al.	Vet Ophthalmol	Intraocular lymphoma is often complicated by uveitis, where inflammation causes trabecular meshwork swelling and iridociliary adhesion, obstructing aqueous drainage and leading to secondary glaucoma.
Mrugacz M et al.	Cells	TNF-α induces ICAM-1 synthesis, promoting leukocyte aggregation and activation, affecting trabecular meshwork function and leading to elevated IOP. IL-1 promotes inflammatory cell migration and aggregation, damaging the trabecular meshwork and optic nerve, and affecting aqueous outflow. VEGF stimulates neovascularization, obstructing aqueous outflow and damaging the optic nerve.
Kapuganti R.S. et al.	Exp Eye Res	TNF-α induces inflammation in the trabecular meshwork, affecting aqueous outflow and exerting toxicity on retinal ganglion cells. IL-6 exacerbates ocular inflammation, interfering with aqueous drainage and damaging the optic nerve. IL-1 alters the structure and function of the trabecular meshwork, increasing IOP and damaging the optic nerve.
Chen J et al.	FASEB J	IL-17A promotes microglial activation and the release of pro-inflammatory cytokines, participating in the regulation of retinal immune responses and RGC cell death via the p38 MAPK pathway. Inhibition of IL-17A can alleviate glaucomatous neuropathy.
Simcoe M.J. et al.	Ophthalmology	The GRM5 gene is involved in inflammatory responses. Myopia may trigger local inflammation and oxidative stress, aligning with the neuroinflammatory mechanisms of glaucoma. It is speculated that TNF-α, IL-1, and IL-6 may damage retinal ganglion cells, interfere with aqueous drainage, and affect optic nerve function.
Chou T.H. et al.	Int J Mol Sci	Early elevation of pro-inflammatory cytokines (specific factors not identified) is speculated to involve TNF-α, IL-1, and IL-6, influencing the neuroinflammatory process in glaucoma.
Jonas J.B. et al.	PLoS One	Using hs-CRP as a systemic inflammation marker, no significant association was found between glaucoma and hs-CRP. Combined with other studies, common glaucoma-related inflammatory factors include TNF-α, IL-1, and IL-6.
Donkor N et al.	Antioxidants (Basel)	CIH leads to increased retinal TNF-α, activating related pathways and triggering inflammatory responses that damage retinal ganglion cells, promoting glaucoma progression.
Nezu N et al.	Ophthalmology	MCP-1, IL-6, and angiogenin are important immune mediators in predicting primary open-angle glaucoma, participating in related pathological processes, though specific mechanisms require further study.
Wang H.N. et al.	Neural Regen Res	TNF-α regulates L- and T-type Ca²^+^ currents and modulates Ca²^+^ channel subunit expression, participating in the apoptosis of RGCs in glaucoma.
Azbukina N.V. et al.	Biology (Basel)	Arachidonic acid can metabolize into inflammation-related substances, participating in the pathological process of glaucoma. Lyso-PAF may convert to PAF, involved in inflammation. Both may interact with inflammatory factors such as TNF-α, IL-1, IL-6, and IFN-γ, influencing the development of glaucoma inflammation.
Yun S et al.	Vet Ophthalmol	In PACG, inflammation-related proteins and pathways are upregulated. SPP1 promotes the secretion of inflammatory factors such as IL-6 and TNF-α, while PGLYRP2 induces the production of IL-1 and IL-6, participating in the inflammatory response in glaucoma.
Frolov M.A. et al.	Vestn Oftalmol	In the high IOP group, concentrations of IL-6 and others are significantly upregulated, with IL-6 positively correlated with high IOP. Anti-MBP antibodies may activate immune cells to release inflammatory factors such as TNF-α, IL-1, and IL-6, participating in the pathological process of glaucoma.
Cao L et al.	Int J Physiol Pathophysiol Pharmacol	Early changes in retinal immune responses in glaucoma. Rac1 may regulate the expression and release of inflammatory factors via the MAPK pathway, suggesting that TNF-α, IL-1, and IL-6 participate in early retinal pathological processes.
Elshahaly M et al.	Curr Rheumatol Rev	In Behçet’s disease patients with uveitis, females have high levels of acute-phase reactants ESR and CRP, suggesting a link to the inflammatory process in glaucoma. Inflammatory factors such as TNF-α, IL-1, and IL-6 may also influence the onset and progression of glaucoma.
Wang C.Y. et al.	Ophthalmic Res	IL-6 is associated with the severity of normal-tension glaucoma, regulating immune cell activity and affecting the survival or death of retinal ganglion cells. The IL-6 (-174) locus gene polymorphism influences the onset and progression of glaucoma.
Reinehr S et al.	Front Cell Neurosci	In autoimmune glaucoma models, complement system activation is observed with increased deposition of C3 and MAC, suggesting that intraocular inflammation plays a role in glaucomatous neurodegeneration. TNF-α, IL-1, and IL-6 may influence C3 and MAC, participating in the inflammatory process of glaucoma.

VEGF, Vascular Endothelial Growth Factor; IL, Interleukin; TNF-α, Tumor Necrosis Factor-alpha; lncRNA, Long Non-Coding RNA; ceRNA, Competing Endogenous RNA; ICAM-1, Intercellular Adhesion Molecule 1; RGC, Retinal Ganglion Cell; MAPK, Mitogen-Activated Protein Kinase; GRM5, Glutamate Metabotropic Receptor 5; hs-CRP, High-Sensitivity C-Reactive Protein; CIH, Chronic Intermittent Hypoxia; MCP-1, Monocyte Chemoattractant Protein-1; PAF, Platelet-Activating Factor; IFN-γ, Interferon-gamma; PACG, Primary Angle, Closure Glaucoma; SPP1, Secreted Phosphoprotein 1; PGLYRP2, Peptidoglycan Recognition Protein 2; MBP, Myelin Basic Protein; Rac1, Ras-related C3 botulinum toxin substrate 1; ESR, Erythrocyte Sedimentation Rate; CRP-C, Reactive Protein; C3, Complement Component 3; MAC, Membrane Attack Complex.

## 3 The Influence of inflammation on the development and progression of glaucoma

### 3.1 Tumor necrosis factor-α

#### 3.1.1 Impact on trabecular meshwork function

TNF-α induces the synthesis of intercellular adhesion molecule-1 (ICAM-1), promotes the abnormal aggregation and activation of white blood cells in ocular tissues, triggers an inflammatory response (11), affects the normal function of the trabecular meshwork, and impedes the outflow of aqueous humor (5), leading to an increase in intraocular pressure.

#### 3.1.2 Damage to retinal ganglion cells

It activates inflammatory cells, releases toxic substances, and disrupts the microenvironment of retinal ganglion cells. TNF-α induces the expression of apoptosis-related proteins, promotes cell apoptosis, and affects the function of the optic nerve (11). By regulating the Glutamate receptor subunit A2 (GluA2) subunit of the α-Amino-3-hydroxy-5-methyl-4-isoxazolepropionic acid (AMPA) receptor, it increases the intracellular Calcium ion (Ca²^+^) concentration, enhances the current mediated by Voltage-gated sodium channel 1.6, raises cell excitability, and promotes the apoptosis of retinal neurons (16).

#### 3.1.3 Regulation of calcium channel currents

In a rat model of chronic ocular hypertension in glaucoma, TNF-α inhibits L-type calcium currents, enhances T-type calcium currents, regulates the expression of Ca²^+^ channel subunits, affects intracellular Ca²^+^ homeostasis, and participates in the apoptotic process of retinal ganglion cells in glaucoma (16).

#### 3.1.4 Involvement in the inflammatory cascade

Metabolites of arachidonic acid can induce cells to produce TNF-α. TNF-α activates inflammatory cells, releases other inflammatory mediators, amplifies the inflammatory response, and drives the progression of glaucoma. Lyso-PAF may be converted into PAF, indirectly prompting cells to release TNF-α and participating in the inflammatory process of glaucoma (17).

#### 3.1.5 Activation of the complement system

TNF-α activates immune cells, promotes the synthesis and release of Complement component 3 (C3), enhances the expression of complement receptors on the surface of immune cells, activates the complement cascade, and increases the generation of the membrane attack complex (MAC). Complement activation, particularly via the ​lectin pathway, drives early neuroinflammation in glaucoma. In experimental models, ​ Mannan-binding lectin-associated serine protease​ 2 (MASP2)​ upregulation triggers C3 cleavage, leading to ​C3 deposition​ and​ MAC formation in the retina and optic nerve, which directly damages retinal ganglion cells (RGCs) and oligodendrocytes (7). C3a/C5a fragments recruit pro-inflammatory microglia, amplifying TNF-α/IL-1β release and disrupting the blood-retinal barrier. Hypoxia-inducible Factors-1α​(HIF-1α) (15) and genetic variants like ​IL-6 (-174C)​​ (22) further synergize with complement to exacerbate oxidative stress and neurodegeneration.

### 3.2 Interleukin-6

#### 3.2.1 Interference with aqueous humor drainage

IL-6 promotes the activation and proliferation of immune cells, exacerbates the ocular inflammatory response. It regulates the cytokine network, affects the functions of trabecular meshwork cells and the metabolism of the extracellular matrix, interferes with the normal drainage of aqueous humor, and participates in the process of increasing intraocular pressure (6, 22).

#### 3.2.2 Damage to the optic nerve

IL-6 alters the permeability of ocular blood vessels, leading to tissue edema and affecting the normal physiological functions of the eyes. It acts synergistically with other inflammatory factors (10) to damage the optic nerve and accelerate the deterioration of glaucoma.

#### 3.2.3 Involvement in the disease process

IL-6 is an important immune mediator in the prediction model of primary open-angle glaucoma. In normal-tension glaucoma (NTG), the serum IL-6 level is related to the disease severity (22). The serum IL-6 level in advanced-stage patients is higher than that in early-and mid-stage patients. It may affect the survival or death of retinal ganglion cells and interfere with the normal metabolism and functions of intraocular tissues. The gene polymorphism at the IL-6 (-174) locus is related to some clinical indicators of NTG patients, affecting the expression level or functional activity of IL-6, and thus influencing the occurrence and development of glaucoma.

#### 3.2.4 Active the complement system indirectly

IL-6 promotes B cell proliferation and differentiation, leading to increased antibody production. These antibodies then activate the classical complement pathway by forming immune complexes that bind Complement component 1q (C1q). IL-6 also enhances vascular endothelial cell permeability through intracellular signaling pathways, facilitating complement component extravasation into ocular tissues (7).

### 3.3 Interleukin-1

#### 3.3.1 Triggering the inflammatory response

IL-1 promotes the migration and aggregation of inflammatory cells to sites such as the trabecular meshwork and the optic nerve, triggering a local inflammatory response. It releases multiple inflammatory mediators, which damage the trabecular meshwork tissue and the optic nerve, and affect the normal drainage of aqueous humor and the function of the optic nerve (10, 13, 14, 17).

#### 3.3.2 Interfering with aqueous humor drainage

IL-1 stimulates immune cells, putting the ocular tissues in an inflammatory state. It interferes with the metabolism and functions of trabecular meshwork cells, increases the resistance to aqueous humor outflow, and raises the intraocular pressure (5). Additionally, it affects the synthesis and degradation of the extracellular matrix, alters the structure of the trabecular meshwork, and impedes the drainage of aqueous humor (11).

#### 3.3.3 Amplifying the inflammatory cascade

IL-1 is released in response to the abnormal pathological changes in the eyes of patients with pseudoexfoliation syndrome. It activates inflammatory cells, attracts more immune cells to migrate to the ocular inflammatory sites, and amplifies the inflammatory cascade (11). It also changes the structure and function of the trabecular meshwork, reducing its ability to drain aqueous humor and increasing the intraocular pressure. Moreover, it induces cells to produce other inflammatory mediators and cytokines, disrupting the microenvironment of ocular tissues and damaging the optic nerve (5).

#### 3.3.4 Activating the complement system

IL-1 stimulates ocular cells and immune cells, upregulates the expression of C3, regulates the activity of related proteins in the complement activation pathway, promotes the cleavage and activation of C3, and increases the formation of the MAC. It enhances the chemotaxis of inflammatory cells, amplifies the inflammatory response, and exacerbates the damage of ocular tissues by C3 and MAC (7, 20).

### 3.4 Vascular endothelial growth factor

In neovascular glaucoma (NVG), an increase in VEGF levels raises vascular permeability, causing substances within the blood vessels to leak. It stimulates the division of endothelial cells and promotes neovascularization. The newly formed blood vessels are structurally unstable and prone to leakage, leading to edema of intraocular tissues, the growth of fibrovascular membranes, obstruction of aqueous humor outflow, an increase in intraocular pressure, and further damage to the optic nerve (8). In the highintraocular-pressure group after vitrectomy for retinal detachment, the VEGF concentration is significantly upregulated, suggesting a possible association between VEGF and intraocular inflammation as well as the development of glaucoma (19).

### 3.5 Monocyte chemoattractant protein-1

Monocyte chemoattractant protein-1 (MCP-1), also known as C-C chemokine ligand 2 (CCL2), belongs to the chemokine family. MCP-1 plays a crucial role in the physiological and pathological processes of the eye, especially in the occurrence and development of primary open-angle glaucoma (POAG). MCP-1 is an important immune mediator in predicting primary open-angle glaucoma. It is an indicator of the ocular inflammatory state, capable of recruiting immune cells, promoting the inflammatory response, and participating in the pathological process of glaucoma. However, its specificity is relatively low (6).

### 3.6 Angiogenin

Angiogenin is an important immune mediator in predicting primary open-angle glaucoma and is related to the process of ocular angiogenesis. It is closely associated with ocular angiogenesis. When abnormal, it can lead to disordered angiogenesis. It may play a significant role in the development of glaucoma by causing tissue edema, regulating inflammation, affecting the function of the trabecular meshwork, and so on (6).

### 3.7 Interleukin-17A

IL-17A is significantly upregulated in the retinas of mice with chronic ocular hypertension. It promotes the activation of microglia and the release of pro-inflammatory cytokines and enhances the phenotypic transformation of activated microglia from the M2 type in the early stage to the M1 type in the late-stage glaucoma retina. It promotes the activation of retinal microglia through the p38 Mitogen-Activated Protein Kinase (MAPK) signaling pathway and participates in regulating the retinal immune response and the death of retinal ganglion cells in experimental glaucoma. Inhibiting IL-17A can reduce the loss of retinal ganglion cells and improve axonal quality and the performance of flash visual evoked potential, which is helpful for alleviating glaucomatous neuropathy. Thus, it is an innovative target for glaucoma treatment strategies (12).

### 3.8 Interferon-γ

IFN-γ may be induced and produced during the immune-inflammatory response triggered by arachidonic acid. It activates immune cells such as macrophages, enhances their killing activity and the ability to release inflammatory mediators, regulates the immune response, indirectly affects the survival and function of retinal ganglion cells, and participates in the chronic inflammatory process of glaucoma. Lyso-PAF may indirectly affect the production and release of IFN-γ by regulating the functions of immune cells, thus influencing the inflammatory state of glaucoma (17).

### 3.9 Secreted phosphoprotein 1 (SPP1, also known as osteopontin)

Osteopontin, namely, SPP1, is of great significance in the occurrence and development of glaucoma. It can bind to multiple receptors on the cell surface, attract the aggregation of immune cells, and promote the secretion of inflammatory factors such as IL-6 and TNF-α, thereby activating the inflammatory response (23). In the pathological process of primary angle-closure glaucoma, this inflammatory response interferes with the normal physiological functions of the eye and promotes the progression of the disease. Animal experimental studies have found that SPP1 is significantly increased in the aqueous humor of dogs with a primary angle-closure glaucoma (PACG) model (10).

### 3.10 Peptidoglycan recognition protein 2

PGLYRP2 can recognize bacterial peptidoglycan, activate downstream immune signaling pathways, and promote the massive production of inflammatory factors such as IL-1 and IL-6, triggering an inflammatory response. These inflammatory factors damage the normal structure and function of intraocular tissues, interfere with the drainage of aqueous humor, participate in the inflammatory response of glaucoma, and drive the progression of the disease (10, 24).

## 4 Discussion

Glaucoma, a key eye disease causing irreversible vision impairment worldwide, has a prevalence that is constantly rising with the aging of the population. It imposes a heavy burden on patients, families, and society (25, 26). In the past, glaucoma was often considered a disease caused by congenital abnormalities, while the impact of inflammatory factors during its pathogenesis was overlooked. In recent years, remarkable progress has been made in the research on inflammatory factors in the intraocular fluid of glaucoma patients. This review retrieved relevant articles from the PubMed database, and after screening, 19 articles were selected to deeply explore the mechanism of action of inflammatory factors in the occurrence and development of glaucoma. The study found that the levels of multiple inflammatory factors in the intraocular fluid of glaucoma patients are abnormal, and the inflammatory response plays a crucial role in the pathogenesis of glaucoma. Meanwhile, we made a comparison table for the abnormal cytokine expressions of different glaucoma subtypes, as detailed in **Table 2**.

**Table 2:** Abnormal cytokine expressions of different glaucoma subtypes.

Glaucoma Subtype	​Significantly Elevated Cytokines​	​Significantly Reduced Cytokines​	Pathogenic Mechanisms	​References​
​Neovascular Glaucoma (NVG)​​	VEGF↑, IL-6↑, TNF-α↑	PEDF↓	Ischemia-driven angiogenesis Fibrovascular membrane obstruction	(8, 19)
​Primary Open-Angle Glaucoma (POAG)​​	MCP-1↑, IL-6↑, IL-17a↑	–	Trabecular meshwork inflammation Microglial activation via p38 MAPK	(6, 12, 16)
Primary Angle-Closure Glaucoma (PACG)​​	IL-6↑, SPP1↑	–	Uveoscleral outflow obstruction Macrophage recruitment	(10, 18)
​Normal-Tension Glaucoma (NTG)​​	IL-6↑ (in advanced stages), TNF-α↑	–	IL-6(-174C) polymorphism Complement-mediated neurodegeneration	(7, 22)
​Pseudoexfoliation	IL-1β↑, TNF-α↑	Clusterin↓	Oxidative protein aggregation Lysophospholipid dysregulation	(11, 13)
​OSA-Associated Glaucoma​	HIF-1α↑, 8-OHdG↑	SOD2↓	Chronic hypoxia-reoxygenation injury Mitochondrial ROS overproduction	(15)

VEGF, Vascular Endothelial Growth Factor; IL, Interleukin; TNF-α, Tumor Necrosis Factor-alpha; PEDF, Pigment Epithelium-Derived Factor; MCP, Monocyte Chemoattractant Protein; SPP1, Secreted Phosphoprotein 1; 8-OHdG, 8-Hydroxy-2’-deoxyguanosine; HIF, Hypoxia-Inducible Factor; SOD, Superoxide Dismutase; OSA, obstructive sleep apnea.

"↑" indicates an increase in the concentration of the labeled cytokine, while "↓" indicates a decrease in the concentration of the labeled cells.

Evidence reveals distinct cytokine signatures across glaucoma subtypes, offering diagnostic and therapeutic implications. In NVG, VEGF-driven angiogenesis dominates pathological changes (27), suggesting anti-VEGF biologics as a priority intervention. POAG exhibits elevated MCP-1 and angiogenin levels, indicating chronic leukocyte infiltration (6). IL-6 shows higher aqueous levels in POAG and NTG compared to controls (28), correlating with optic neurodegeneration severity. These subtype-cytokine associations underscore the necessity for personalized therapeutic strategies targeting dominant inflammatory pathways, which may improve treatment precision compared to conventional IOP-lowering approaches.

Among numerous inflammatory factors, TNF-α, IL-6, and IL-1 are particularly critical. TNF-α can affect the function of the trabecular meshwork, damage retinal ganglion cells, regulate calcium channel currents, participate in the inflammatory cascade, and activate the complement system. IL-6 interferes with the drainage of aqueous humor, damages the optic nerve, participates in the disease process, and regulates the complement system. IL-1 triggers the inflammatory response, interferes with aqueous humor drainage, amplifies the inflammatory cascade, and activates the complement system. In addition, multiple inflammatory factors such as VEGF and MCP-1 also play roles to varying degrees in the pathological process of glaucoma.

In addition to the content we retrieved, Emerging evidence highlights additional inflammatory mechanisms in glaucoma pathogenesis. Microglial activation initiates neuroinflammation by releasing reactive oxygen species (ROS) and pro-inflammatory cytokines, accelerating RGC apoptosis (29). Oxidative stress disrupts mitochondrial dynamics through impaired mitophagy, exacerbating RGC vulnerability to intraocular pressure fluctuations (30, 31). Mitochondrial dysfunction manifests as reduced Adenosine Triphosphate (ATP) production and increased cytochrome c release, activating caspase-dependent apoptotic pathways (32, 33). These mechanisms synergistically interact with cytokine networks, forming a self-amplifying inflammatory cascade.

Recent evidence highlights the NACHT, LRR and PYD domains-containing protein 3 (NLRP3) inflammasome as a critical mediator bridging biomechanical stress and neuroinflammation in glaucoma. Elevated IL-1β colocalizes with NLRP3+ astrocytes at the optic nerve head, where ATP release from mechanically stressed RGCs triggers Purinergic receptor P2X7-dependent NLRP3 oligomerization and caspase-1 activation (34). Concurrently, Toll-like Receptor 4 (TLR4) upregulation in glial cells enables the detection of damage-associated molecular patterns, driving NF-κB-mediated TNF-α production through Myeloid Differentiation Primary Response 88 (MyD88) signaling (35, 36). Notably, MCC950 (NLRP3 inhibitor) and TAK-242 (TLR4 antagonist) demonstrate neuroprotection in preclinical models by suppressing Apoptosis-associated Speck-like protein containing a CARD (ASC) speck formation and microglial reactivity. These findings position innate immune sensors as promising therapeutic targets for glaucoma-related neurodegeneration.

At the same time, emerging evidence highlights advanced glycation end-products (AGEs) and their receptor (RAGE) as pivotal contributors to glaucomatous neurodegeneration. The AGEs/RAGE axis promotes oxidative stress and chronic inflammation through sustained NF-κB activation, exacerbating trabecular meshwork dysfunction and retinal ganglion cell apoptosis (37). AGEs accumulate in glaucomatous optic nerve heads and may interact synergistically with TNF-α and IL-6 to amplify neuroinflammatory cascades (38). This pathway accelerates extracellular matrix remodeling and enhances vascular permeability, potentially explaining IOP-independent neurodegeneration in normal-tension glaucoma. Future therapeutic strategies targeting AGE-RAGE signaling may complement existing anti-inflammatory approaches.

In glaucoma, emerging anti-inflammatory approaches hold great potential. Some investigational drugs like small-molecule inhibitors target key inflammatory pathways. Currently, there are ongoing clinical trials exploring biologics such as monoclonal antibodies against inflammatory cytokines. These efforts seek to develop more effective therapies by precisely targeting inflammation, which could potentially enhance the current glaucoma treatment strategies (39).

Based on these findings, future glaucoma treatments can be carried out in multiple aspects. In terms of drug therapy, biological agents targeting inflammatory factors can be developed, such as TNF-α inhibitors, IL-6 receptor antagonists, and IL-1 antagonists. In the field of gene therapy, gene editing techniques and RNA interference technologies can be utilized to regulate the expression of inflammation-related genes. Moreover, by using inflammatory factors as biomarkers, personalized and precise treatments can be implemented according to patients’ inflammatory factor profiles and genetic characteristics. However, challenges persist in ocular drug delivery due to blood-retinal barrier restrictions, requiring nanoparticle-based delivery systems for targeted therapy (40). Systemic immunosuppression risks infection exacerbation and requires careful risk-benefit evaluation (41). While animal models demonstrate efficacy, interspecies differences in immune responses may limit clinical translation (42). Gene therapy faces technical hurdles in vector selection and ethical concerns regarding off-target effects (43). Additionally, inequitable healthcare resource allocation, particularly the prohibitive costs of advanced therapies exacerbating health disparities, constitutes a critical socioeconomic and ethical challenge in clinical translation.

This review is of great significance for clinical practice and research. Clinically, it helps clinicians quickly master the cutting-edge knowledge related to inflammatory factors in glaucoma. By monitoring these factors, early diagnosis and treatment of glaucoma can be achieved, providing a theoretical basis for formulating more precise treatment plans. In the research field, it clarifies the deficiencies of existing studies, points the way for subsequent scientific research, and promotes the innovative development of glaucoma treatment. In the future, it is expected that through in-depth research on inflammatory factors, more targeted therapeutic drugs, and intervention measures can be developed, further improving the prognosis of glaucoma patients worldwide and reducing the social burden imposed by glaucoma.

Notably, our reliance on PubMed alone introduces potential selection bias despite its comprehensive biomedical coverage. While MeSH normalization optimized precision, studies excluded other datasets, which may lead to underrepresentation. This methodological constraint necessitates a cautious interpretation of the generalizability of the therapeutic mechanism, particularly regarding non-English evidence.
